# Stimulated Raman histology in the neurosurgical workflow of a major European neurosurgical center — part A

**DOI:** 10.1007/s10143-021-01712-0

**Published:** 2021-12-16

**Authors:** Nicolas Neidert, Jakob Straehle, Daniel Erny, Vlad Sacalean, Amir El Rahal, David Steybe, Rainer Schmelzeisen, Andreas Vlachos, Peter Christoph Reinacher, Volker Arnd Coenen, Boris Mizaikoff, Dieter Henrik Heiland, Marco Prinz, Jürgen Beck, Oliver Schnell

**Affiliations:** 1 Department of Neurosurgery, Medical Center, University of Freiburg, Freiburg, Germany; 2 Microenvironment and Immunology Research Laboratory, Medical Center, University of Freiburg, Freiburg, Germany; 3 Institute of Neuropathology, Faculty of Medicine, University of Freiburg, Freiburg, Germany; 4 Department of Oral and Maxillofacial Surgery, Medical Center, University of Freiburg, Freiburg, Germany; 5 Medical Faculty, Freiburg University, Freiburg, Germany; 6 Department of Neuroanatomy, Institute of Anatomy and Cell Biology, Faculty of Medicine, University of Freiburg, Freiburg, Germany; 7 Center for Basics in NeuroModulation (NeuroModulBasics), Faculty of Medicine, University of Freiburg, Freiburg, Germany; 8 Center Brain Links Brain Tools, University of Freiburg, Freiburg, Germany; 9 Department of Stereotactic and Functional Neurosurgery, Medical Center, University of Freiburg, Freiburg, Germany; 10 Fraunhofer Institute for Laser Technology (ILT), Aachen, Germany; 11 Institute of Analytical and Bioanalytical Chemistry, Ulm University, Ulm, Germany; 12 Hahn-Schickard Institute for Microanalysis Systems, Ulm, Germany; 13 Comprehensive Cancer Center Freiburg (CCCF), Faculty of Medicine and Medical Center, University of Freiburg, Freiburg, Germany; 14 German Cancer Consortium (DKTK), partner site Freiburg, Freiburg, Germany; 15 Signalling Research Centres BIOSS and CIBSS, University of Freiburg, Freiburg, Germany

**Keywords:** Neurosurgery, Stimulated Raman histology, Tissue imaging, Neurooncolocy, Extent of resection

## Abstract

Histopathological diagnosis is the current standard for the classification of brain and spine tumors. Raman spectroscopy has been reported to allow fast and easy intraoperative tissue analysis. Here, we report data on the intraoperative implementation of a stimulated Raman histology (SRH) as an innovative strategy offering intraoperative near real-time histopathological analysis. A total of 429 SRH images from 108 patients were generated and analyzed by using a Raman imaging system (Invenio Imaging Inc.). We aimed at establishing a dedicated workflow for SRH serving as an intraoperative diagnostic, research, and quality control tool in the neurosurgical operating room (OR). First experiences with this novel imaging modality were reported and analyzed suggesting process optimization regarding tissue collection, preparation, and imaging. The Raman imaging system was rapidly integrated into the surgical workflow of a large neurosurgical center. Within a few minutes of connecting the device, the first high-quality images could be acquired in a “plug-and-play” manner. We did not encounter relevant obstacles and the learning curve was steep. However, certain prerequisites regarding quality and acquisition of tissue samples, data processing and interpretation, and high throughput adaptions must be considered. Intraoperative SRH can easily be integrated into the workflow of neurosurgical tumor resection. Considering few process optimizations that can be implemented rapidly, high-quality images can be obtained near real time. Hence, we propose SRH as a complementary tool for the diagnosis of tumor entity, analysis of tumor infiltration zones, online quality and safety control and as a research tool in the neurosurgical OR.

## Introduction

The annual incidence of primary brain tumors in the USA was 23.79 per 100,000 (2013–2017) and the incidence of brain metastases is expected to be approximately four times higher, although recent and robust numbers are currently not available [14]. Neurosurgical tumor resection is among of the main pillars of the neurooncological treatment concepts of brain tumors, and therefore indicated in many such patients at some point in the course of the disease. Maximizing the extent of resection has a beneficial impact on the overall survival of neurooncological patients [3, 13, 17].

 In addition to the surgeon’s anatomical skills and experience, several modalities are commonly used in the operating theater to achieve the goal of gross total resection (GTR) in brain tumor surgery including but not limited to fluorescence-guided surgery after immediate preoperative administration of 5-aminolevulinic acid (5-ALA), which has been shown to increase the extent of resection and subsequently the progression-free survival in glioblastoma patients [19]. However, the use in other brain tumor pathologies may be limited [2] and there is also no clear evidence that either neuronavigation [21] or intraoperative ultrasound [6] is able to maximize the extent of resection. Intraoperative MRI (iMRI) has shown benefits in achieving GTR [16]; however, this modality is not accessible in every institution and implementation in the routine surgical workflow is not without efforts.

Classification and prognostication of brain, skull base, or spinal tumors is still based on histopathological diagnosis, which is the gold standard. In general, conventional intraoperative histopathological diagnosis is based on the interpretation of hematoxylin–eosin (H&E) staining of fast frozen sections. Conventionally, technical assistants and an experienced neuropathologist are needed to process and analyze the tissue. Afterwards, the histopathological findings are reported to the surgical team. This procedure is time consuming and labor intensive. In contrast to oncological surgeries in other disciplines where achievement of tumor-free resection borders is the goal, intraoperative histopathological examination of tumor borders is not regularly performed during neurosurgical resection.

Raman spectroscopy (RS) and in particular stimulated Raman histology (SRH) are innovative techniques that allow for a near to real-time intraoperative tissue analysis [9, 10]. They have the potential to add important information in addition to conventional intraoperative histology. SRH can visualize neuroanatomical structures such as axons, dendrites, and glial processes [9] and is label-free and fast [10]. It has been shown that intraoperative pathological assessment of SRH images is en par to the assessment of standard of care H&E staining of fast frozen sections [5]. The NIO Laser Imaging System (Invenio Imaging Inc., Santa Clara, CA, USA) is a commercially available SRH device for intraoperative application and has recently gained CE-certification.

In this study, we want to present our first experience with intraoperative SRH imaging > 150 resected tissue specimens of 108 neurosurgical patients. We aim at sharing aspects of process optimization and avoidance of methodical pitfalls needed to maximize the information gained by SRH and to explore the potential of intraoperative SRH as a tool for analyzation of tumor infiltration at the border zone to normal tissue, online quality and safety control, and its utility as a research tool in the operating room (OR).

## Materials and methods

### Patients

During a period of 6 weeks, tissue specimens of more than 100 consecutive patients with various CNS lesions and mainly brain or spinal tumors were included in this study. Written informed consent was obtained and intraoperative tissue processing and imaging with the SRH system and the respected workflow were documented and analyzed for feasibility of routine clinical implementation.

### Ethics

The local ethics committee of the University of Freiburg approved data evaluation, imaging procedures, and experimental design (protocol 5565/15). The methods were carried out in accordance with the approved guidelines, with written informed consent obtained.

### Process analysis and workflow description

An initial workflow for intraoperative SRH was based on the recent literature and adapted to our institutional framework conditions. Correct and reasonable workflow steps were perpetuated and unfavorable steps were documented, erased, and replaced by more practical steps as soon as during the next sample acquisition.

### Tissue preparation

**Fig. 1:**
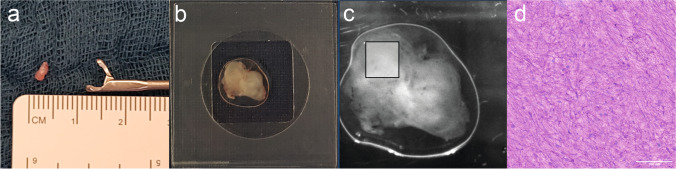
Tissue acquisition is demonstrated. **a** Example of a specimen extracted with a tumor grasping forceps. **b** Compressed tissue on an imaging object carrier, which is subsequently loaded in the NIO Laser Imaging System [9]. A digital overview image of the tissue sample is created (**c**). A section of the tissue was selected for SRH imaging and an excerpt of the generated SRH image is shown (**d**). The length of the scale bar in the lower right corner represents 100 µm

In unformal testing, tissue specimens of 8 mm^3^ (Fig. 1a) were shown to be ideal for SRH imaging. Different forceps and pincers were examined for an optimal tissue extraction. Specimens were placed on a wet cotton polyester gaze (Melolin, Smith & Nephew, London, England) and transported to the central imaging room in the OR. The exact anatomical location of every sample was documented by using the neuronavigation software of either a Stryker®- (Stryker Corporation, Kalamazoo, MI, USA) or Brainlab® system (Brainlab, München, Germany). A written note concerning the anatomical localization was also documented. A corresponding tissue sample of the exact adjacent location was sent for conventional intraoperative histopathology (Fig. 3).

It was of major importance that the resected tissue sample was hardly coagulated with a bipolar and blood poor. However, remaining blood could be reduced by washing the tumor tissue in Ringer’s lactate solution. If tissue specimens larger than 8 mm^3^ were obtained, a scalpel and forceps were used to cut up the tissue. Rigid, bloody, or coagulated parts of the sample were cut away prior to imaging.

### Stimulated Raman histology

Small tissue sections down to 8 mm^3^ were placed on a plastic carrier and were subsequently compressed by a small coverslip to a thickness of 230 µm (Fig. 1b). The carrier was loaded into the NIO Laser Imaging System (Invenio Imaging Inc., Santa Clara, CA, USA). Mostly, multiple 2 × 2 mm areas of the specimens were selected for SRH. The sample holder designed for the use of SRH had a diameter of 16 mm. If a section was rich in axons, dendrites, reactive astrocytes, or other fibrous structures, three-dimensional 0.5 × 0.5 mm imaging of the tissue was performed in 30 sections at a distance of 1 µm between the images.

SRH was performed by using the NIO Laser Imaging System (Invenio Imaging Inc., Santa Clara, CA, USA) according to the manufacturer’s requirements and as previously described by Hollon et al. [9]. Stimulation with a laser was used to image at a tissue depth of 10 µm evaluating two characteristic Raman shifts at 2845 cm^−1^ and 2940 cm^−1^, respectively. The 2845 cm^−1^ signal is characteristic for lipid-rich structures; protein- and DNA-rich patterns are responsible for the 2940 cm^−1^ signal. Multiple line scans with a width of 1000 pixels at a pixel size of 467 nm were imaged as raw SRH data. These SRH images were used to create a virtual H&E-like SRH image by subtraction and assignment via a proprietary lookup table included in the instrument software. The derived false-color H&E-like SRH images were made accessible to the surgeon and later to a board-certified neuropathologist (cf Straehle et al., [18]).

### Tissue storage and possibility of later analysis

The compressed tissue samples were transported to our neurosurgical laboratory, frozen immediately in liquid nitrogen (N_2_), and stored in a − 80 °C freezer (Fig. 3). Therefore, the possibility for later molecular analysis was ensured.

## Results

### Patients and pathologies

In total, tissues from 108 consecutive neurosurgical patients were imaged during a 6-week period. A total of 73 patients underwent surgery for newly diagnosed or recurrent neoplastic diseases. The three most frequent entities were gliomas (*n* = 23), metastases (*n* = 20), and meningeomas (*n* = 11). However, also tissue samples of rare entities were imaged, for example, subependymoma (*n* = 2), epidermoid cysts (*n* = 2), ganglioglioma, or hemangioblastoma (*n* = 1, each). In the non-neoplastic collective (*n* = 35), we analyzed resected tissue like nucleus pulposus or arachnoid cyst wall, which was also helpful to optimize tissue prerequisites for SRH analysis.

Furthermore, imaging of entry cortex from brain tumor surgeries or resected tissue from epilepsy surgeries helped us to understand how to assess and interpret non-tumor infiltrated tissue. In total, 429 SRH images were generated. Table 1 summarizes the investigated tissue samples according to their entity, number of analyzed patients, and respected samples and SRH images per patient.

**Table 1:** Summary of the number of patients listed according to the tissue samples examined. The mean number of samples analyzed per patient and the mean number of SRH images obtained per patient are shown

Entitiy	Number of patients	Samples per patient	SRH images per patient	Tumor (T)/other (O)
Glioma	23	2.2	5.1	T
Metastases	20	1.5	4.3	T
Meningeomas	11	1.2	2.3	T
Schwannoma	2	1.5	3.5	T
Pituitary adenoma	7	1.3	3.3	T
Epidermoid cyst	2	1.5	4	T
Colloid cyst	1	2	9	T
Subependymoma	2	1	3.5	T
Lymphoma	1	1	3	T
Hemangioblastoma	1	2	4	T
Ganglioglioma	1	2	4	T
Neurofibroma	1	1	3	T
Dysembryoplastic neuroepithelial tumor (DNET)	1	1	3	T
Necrosis/reactive tissue	3	2	4.3	T/O
Soft tissue	11	1.5	2.7	O
Intervertebral disc	4	1.3	2.3	O
Epileptic tissue	6	1.4	3	O
Infectious tissue	2	1.5	4	O
Membranes	11	1.4	3.5	O

### Sample acquisition

A tumor grasping forceps (8591A, Karl Storz, Germany) was used for tissue acquisition in microsurgical resection. A standard biopsy forceps was used for stereotactical biopsies (Inomed, Emmendingen, Germany). Especially in diffuse infiltrating tumors, multiple samples were obtained. Therefore, micro- and macroscopically different appearing putative pathological tissue was selected. After compression of the tissue specimen, multiple 2 × 2 mm sections were selected for SRH imaging (Fig. 1c).

**Fig. 2:**
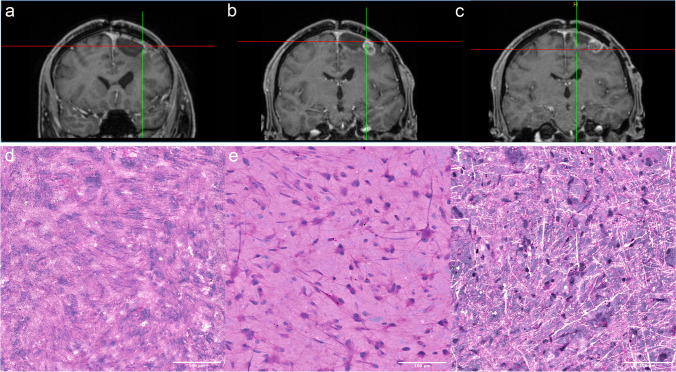
Coronal MRI images (upper row, **a**–**c**) and intraoperative SRH images (lower row, **d**–**f**) from a 49-year-old male patient, who underwent surgery for a recurrent left frontal glioblastoma. **d** The SRH image of contrast-enhancing cyst wall of the previous resection cavity (**a**). **e** A SRH image of the contrast-enhancing solid tumor nodulus (**b**). Infiltration zone (**c**) was also imaged (**f**). The length of the scale bar in the lower right corner represents 100 µm

Figure 2 depicts the possibility to visualize different tumor regions intraoperatively in near real time. The potential discrimination between necrosis, vital tumor core, and infiltration may help the surgeon to optimize the extent of resection.

### Workflow and duration of intraoperative SRH imaging

In total, images from 108 patients were obtained on 29 working days, which lead to a mean volume of 3.72 cases per day (ranging from 1 to 9 cases per day). The average number of images acquired was about 14.8 per day. High-quality virtual quasi-H&E SRH images were robustly generated intraoperatively without any complex additional tissue preparation requirements. Within a few minutes of connecting the device, the first high-quality images could be acquired in a “plug-and-play” manner. The implementation of the system in our department has been uncomplicated and intuitive. The learning curve was steep, and only during the first days of usage, two persons (i.e., a neurosurgical resident and a technician) were needed for tissue preparation, imaging, and for data processing. Subsequently, one neurosurgical resident was sufficient for executing these steps.

### Feasibility and quality control for intraoperative SRH

Rigid samples are less suitable for SRH imaging. Tissue has to be readily compressible to avoid artifact-causing air inclusions. Another problem with stiff specimens was potential glass breakage during the process of tissue compression. We therefore did not manage to image bony structures or stiff connective tissues. For example, it was not possible to image anulus fibrosus of a herniated intervertebral disc, whereas nucleus pulposus was sufficiently soft to be loaded onto the sample carriers (Table 2).

**Table 2:** Summary of results during informal testing of SRH imaging for samples of different histological origins or after pretreatment

	Brain tumor tissue	Anulus fibrosus	Nucleus pulposus	Paravertebral soft tissue	Bone lesion	Formaldehyde fixed tissue	Thawed after cryo-conservation
Suitable for SRH	X		X	X		X	X
**Not** suitable for SRH		X			X		

In order to use SRH for visualization of bacterial infections, we faced the problem that the sample carrier is not designed to image fluids but to image intact tissue, as the system needs a reference for the autofocus with a high cell density. Therefore, we have prepared an emulsion of the centrifuged fluid — for example, CSF — of a patient with fulminant bacterial meningitis or pus from abscess drainage in a prewarmed low melting agarose gel. The emulsion was then loaded onto the sample carrier. However, to date, it was not possible to display immune cells in the emulsion or even bacteria. This may be due to the fact that high-quality imaging with the SRH system is optimized for a sampling depth of 10 µm (Table 2).

### Data processing of SRH images

**Fig. 3:**
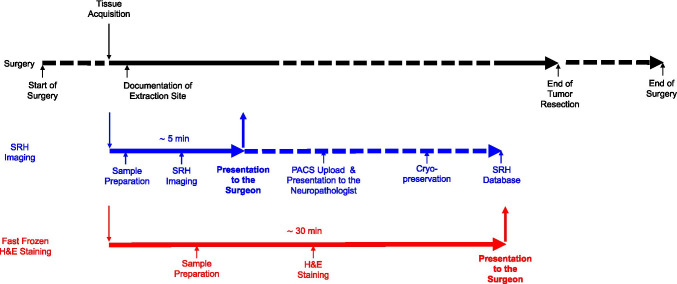
Workflow of SRH imaging in neurosurgery: The extraction site of the tissue was documented on the basis of neuronavigational data shortly after the tissue acquisition. The specimens were then transported to our central SRH imaging room and prepared as mentioned in the “Materials and methods” section. The exact adjacent location was sent to the Institute of Neuropathology for conventional intraoperative fast-frozen H&E staining. After SRH imaging, the data was presented to the surgeon via a tablet with Wi-Fi connection. We implemented the possibility to upload the SRH images onto our PACS system. Samples were cryopreserved for later analysis

SRH images were saved as DICOM files. In order to facilitate intraoperative accessibility of the SRH images for the neurosurgeon and neuropathologist, we only recently set a pipeline to upload and store the SRH files into our clinic’s imaging system IMPAX VIII (Agfa, Mortsel, Belgium) (Fig. 3). This opens new possibilities for interdisciplinary communication, as the pathologist is able to assess the SRH images remotely. It also allows faster and more convenient intradisciplinary communication, as more experienced colleagues may readily be consulted via a digital pathway in difficult cases.

### High-throughput adaptations

Since we acquired more than 14 images from more than 3 patients on a daily average, we had to adapt the workflow for the associated high-throughput demands. The concept of the SRH system suggests the use directly in the OR. In our experience, tissue acquisition occurred rather simultaneously in different concomitant operations. Therefore, it would be bothersome to transfer the imaging system fast enough. To optimize the workflow for our needs and adapt it to our OR infrastructure, we have placed the device at a fixed location in a research laboratory co-located at the OR floor and the samples were transferred directly after acquisition into this laboratory for further processing. The SRH images were initially presented to the surgeon via a tablet with Wi-Fi connection, which was recently complemented by connection of the device to our clinic’s imaging system IMPAX VIII (Agfa, Mortsel, Belgium) (Fig. 3).

Due to the high sample volume, meticulous and systematic documentation on paper and digitally was necessary in order to prevent any loss of information for a specific sample. The documentation of the distinct extraction point of the tissue in the neuronavigation software proved time consuming in the beginning.

#### Discussion

Standard-of-care intraoperative histopathology is the gold standard and helpful for operative decision-making in neurosurgical oncology. Nevertheless, it is time consuming and laborious. SRH is a new and innovative technique for near real-time intraoperative tissue imaging. It is easy to implement into the neurosurgical workflow and allows to rapidly obtain valuable and additional information relevant for the neurosurgeon. Therefore, SRH may indeed become a game changer for intraoperative tissue diagnostics during neurosurgical tumor resection, even if several technical alternatives for SRH in the field of neurosurgery already exist.

Next to sampling with subsequent analysis, there is the possibility of using a handheld probe for intraoperative tissue analysis on the basis of Raman spectroscopy [4,21]. However, this technique has several disadvantages, as the interpretation of the Raman spectra is solely possible via computational algorithms. A near real-time user-based analysis, which is one of the advantages of SRH, is not possible for the handheld probe and also not for table-top Raman spectroscopy [15]. Moreover, white-light microscopy has to be paused while using a handheld Raman spectroscopy probe, which leads to a disruption of the neurosurgical workflow. Last but not least, the technique is easily disturbed by small movements.

Another alternative for intraoperative digital histopathology is fluorescein-assisted confocal laser endomicroscopy — the Convivo® System (Carl-Zeiss-Jena, Jena, Germany) [8]. This system uses a handheld confocal laser endomicroscopy probe, which allows image acquisition in the brain without resection of the tissue. Due to the nature of confocal microscopy, it allows to capture images within a z-stack of 30-µm depth. However, the intravenous application of fluorescein as a dye is required to this date. The use of the Convivo®-device ex vivo was shown to be non-inferior to the gold standard [1] and similar results may be expected also in vivo.

A major application of SRH is obtaining diagnostic information about the entity of resected putative pathological tissue. As described above, SRH images have now been integrated as DICOM files in our clinic’s imaging system IMPAX VIII immediately after imaging. Therefore, neuropathological assessment is possible at yet unprecedented speed. Previous studies [5] and very recent studies from our group (cf. Straehle et al., [18]) showed a non-inferiority of the assessment of SRH images by a board-certified neuropathologist compared to the assessment of H&E-stained frozen sections.

Another potential use case of Raman-based methodologies is the detection of metabolic changes in tumor tissue, which could be a surrogate parameter for specific mutations. The relevance of molecular diagnostics in brain tumors is high, as the new 2021 WHO classification of tumors of the central nervous system places a higher priority on molecular changes than ever before [12]. Raman spectroscopy in general has shown to be a precise and reliable tool to determine changes at a molecular level. For example, the non-invasive Raman-based measurements of blood glucose levels are now established [11]. IDH mutations are also causing metabolic changes, which are detectable by Raman spectroscopy [20]. In future, research on modifications of hardware and software could also facilitate the intraoperative direct prediction of crucial molecular changes in the tumor. 

SRH imaging may also be useful as an additional tool to achieve a greater extent of resection in diffusely infiltrating brain tumors. However, one should consider that there are differences between a real H&E image and the virtual H&E-like SRH image. For example, protein-rich extracellular fibers are usually red in the conventional H&E imaging, but may appear eosinophil in the virtual H&E-like SRH image. In order to use SRH as a robust modality to determine tumor histology or tissue diagnosis at the suspected tumor border, it is inevitable to fully understand the limitations and alternative illustration of SRH in comparison to classical staining in health and disease. These are also absolute prerequisites in order to use the SRH technology as an intraoperative tool for improvement of the extent of resection. One problem is the fact that the information provided by SRH imaging of white matter is very sparse (cf. Straehle et al., [18]). In turn, the information-richness of SRH goes far beyond conventional staining. Given the molecular complexity of tissues, additional molecular markers may be identified and allocated via their characteristic vibrational signatures (i.e., at different Raman shifts). Thus, additional false-color maps illustrating particular molecular distributions or the location of specific molecular changes at the same sample may yield multiple yet inherently correlated images each highlighting a particular molecular feature still label-free.

The near real-time character of tissue analysis predestines SRH imaging for quality control of intraoperative biopsies. Still, if small lesions are resected or are subject to stereotactic biopsy, it is of utmost importance to increase the diagnostic yield of the specimen. Using SRH, an additional quality control step is implemented in addition to the standard of care intraoperative neuropathological assessment by H&E staining of frozen sections without disturbance of the neurosurgical workflow. One technical limitation of SRH is that the examined tissue has to be readily and uniformly compressible. Therefore, at the moment, it is not possible to image stiff specimen, which reduces the applicability of this method. On the other hand, SRH is not only limited to the field of neurosurgery. Other applications for example in Head and Neck surgery were also reported [7]. In general, every tissue type that is compressible is suitable for analysis via this technique.

Our study shows that use of SRH imaging in a major European neurooncological center is feasible although the measures taken for this cause are not without drawbacks. Using the imaging system for multiple ORs can lead to delays in tissue processing, if the system is already imaging. This has never exceeded more than a few minutes and did not lead to noticeable lower quality of SRH imaging, but it may lead to suboptimal tissue handling in these exceptional cases.

Adaptations to the high throughput application of SRH imaging proved themselves effective. Nevertheless, some difficulties regarding the exact registration and documentation of the site of tissue acquisition remain. This issue refers to the tissue acquisition, for example, in a brain tumor resection but not for stereotactic biopsy. Of course, annotation points were ideally inserted for every sample in the MRI 3D dataset of the neuronavigation system. Yet, system inherent factors like inaccuracies of the initial patient registration or the selection of the exact extraction localization with the pointer are only minor problems. Brain shift effects on the accuracy of the neuronavigation system prevent a precise and valid documentation of the exact tissue extraction points.

## Conclusions

Our experience shows that the implementation of intraoperative SRH is indeed feasible and beneficial, and is readily and rapidly adaptable to the local conditions and workflows, if certain prerequisites are considered. SRH is not a replacement of classical intraoperative neuropathological assessment, but rather a useful complementary addition for tissue analysis in neurooncological surgeries. We firmly believe that SRH has the potential to evolve into a useful tool to guide the extent of resection in the operating room (OR). Next to the practical utility in routine usage, it is also a valuable method for future neurosurgical studies and intensifies our understanding of the OR as part of a research laboratory. Augmented by deep learning/machine learning/AI-based automated analysis and classification, we are convinced that this technology will pave the way towards label-free digital histopathology in a wide range of OR scenarios and settings.

## Data Availability

Not applicable.

## References

[1] Acerbi F, Pollo B, De Laurentis C, Restelli F, Falco J, Vetrano IG, Broggi M, Schiariti M, Tramacere I, Ferroli P, DiMeco F (2020). Ex vivo fluorescein-assisted confocal laser endomicroscopy (CONVIVO® System) in patients with glioblastoma: results from a prospective study. Front Oncol.

[2] Boschi A, Della Puppa A (2019). 5-ALA fluorescence on tumors different from malignant gliomas. Review of the literature and our experience. J Neurosurg Sci.

[3] Brown TJ, Brennan MC, Li M, Church EW, Brandmeir NJ, Rakszawski KL, Patel AS, Rizk EB, Suki D, Sawaya R, Glantz M (2016). Association of the extent of resection with survival in glioblastoma: a systematic review and meta-analysis. JAMA Oncol.

[4] Desroches J, Jermyn M, Mok K, Lemieux-Leduc C, Mercier J, St-Arnaud K, Urmey K, Guiot M-C, Marple E, Petrecca K, Leblond F (2015). Characterization of a Raman spectroscopy probe system for intraoperative brain tissue classification. Biomed Opt Express.

[5] Eichberg DG, Shah AH, Di L, Semonche AM, Jimsheleishvili G, Luther EM, Sarkiss CA, Levi AD, Gultekin SH, Komotar RJ, Ivan ME (2019). Stimulated Raman histology for rapid and accurate intraoperative diagnosis of CNS tumors: prospective blinded study. J Neurosurg.

[6] Fountain DM, Bryant A, Barone DG, Waqar M, Hart MG, Bulbeck H, Kernohan A, Watts C, Jenkinson MD (2021) Intraoperative imaging technology to maximise extent of resection for glioma: a network meta-analysis. Cochrane Database Syst Rev 1:CD013630. 10.1002/14651858.CD013630.pub2 10.1002/14651858.CD013630.pub2 PMC8094975 33428222

[7] Hoesli RC, Orringer DA, McHugh JB, Spector ME (2017). Coherent Raman scattering microscopy for evaluation of head and neck carcinoma. Otolaryngol-Head Neck Surg Off J Am Acad Otolaryngol-Head Neck Surg.

[8] Höhne J, Schebesch K-M, Zoubaa S, Proescholdt M, Riemenschneider MJ, Schmidt NO (2021). Intraoperative imaging of brain tumors with fluorescein: confocal laser endomicroscopy in neurosurgery. Clinical and user experience Neurosurg Focus.

[9] Hollon T, Orringer DA (2021). Label-free brain tumor imaging using Raman-based methods. J Neurooncol.

[10] Hollon TC, Pandian B, Adapa AR, Urias E, Save AV, Khalsa SSS, Eichberg DG, D’Amico RS, Farooq ZU, Lewis S, Petridis PD, Marie T, Shah AH, Garton HJL, Maher CO, Heth JA, McKean EL, Sullivan SE, Hervey-Jumper SL, Patil PG, Thompson BG, Sagher O, McKhann GM, Komotar RJ, Ivan ME, Snuderl M, Otten ML, Johnson TD, Sisti MB, Bruce JN, Muraszko KM, Trautman J, Freudiger CW, Canoll P, Lee H, Camelo-Piragua S, Orringer DA (2020). Near real-time intraoperative brain tumor diagnosis using stimulated Raman histology and deep neural networks. Nat Med.

[11] Kang JW, Park YS, Chang H, Lee W, Singh SP, Choi W, Galindo LH, Dasari RR, Nam SH, Park J, So PTC (2020) Direct observation of glucose fingerprint using in vivo Raman spectroscopy. Sci Adv 6:eaay5206. 10.1126/sciadv.aay5206 10.1126/sciadv.aay5206 PMC6981082 32042901

[12] Louis DN, Perry A, Wesseling P, Brat DJ, Cree IA, Figarella-Branger D, Hawkins C, Ng HK, Pfister SM, Reifenberger G, Soffietti R, von Deimling A, Ellison DW (2021). The 2021 WHO classification of tumors of the central nervous system: a summary. Neuro-Oncol.

[13] Olesrud IC, Schulz MK, Marcovic L, Kristensen BW, Pedersen CB, Kristiansen C, Poulsen FR (2019). Early postoperative MRI after resection of brain metastases-complete tumour resection associated with prolonged survival. Acta Neurochir (Wien).

[14] Ostrom QT, Patil N, Cioffi G, Waite K, Kruchko C, Barnholtz-Sloan JS (2020) CBTRUS statistical report: primary brain and other central nervous system tumors diagnosed in the United States in 2013–2017. Neuro-Oncol 22:iv1–iv96. 10.1093/neuonc/noaa200 10.1093/neuonc/noaa200 PMC7596247 33123732

[15] Pohling C, Bocklitz T, Duarte AS, Emmanuello C, Ishikawa MS, Dietzeck B, Buckup T, Uckermann O, M.d GS, Kirsch M, Schmitt M, Popp J, Motzkus M,  (2017). Multiplex coherent anti-Stokes Raman scattering microspectroscopy of brain tissue with higher ranking data classification for biomedical imaging. J Biomed Opt.

[16] Senft C, Bink A, Franz K, Vatter H, Gasser T, Seifert V (2011). Intraoperative MRI guidance and extent of resection in glioma surgery: a randomised, controlled trial. Lancet Oncol.

[17] Smith JS, Chang EF, Lamborn KR, Chang SM, Prados MD, Cha S, Tihan T, Vandenberg S, McDermott MW, Berger MS (2008). Role of extent of resection in the long-term outcome of low-grade hemispheric gliomas. J Clin Oncol Off J Am Soc Clin Oncol.

[18] Straehle J, Erny D, Neidert N, Heiland DH, El Rahal A, Sacalean V, Steybe D, Schmelzeisen R, Vlachos A, Mizaikof B, Reinacher PC, Coenen VA, Prinz M, Beck J, Schnell O (2021) Neuropathological interpretation of stimulated Raman histology images of brain and spine tumors: part B (in press) 10.1007/s10143-021-01711-1 PMC8976804 34890000

[19] Stummer W, Pichlmeier U, Meinel T, Wiestler OD, Zanella F, Reulen H-J, ALA-Glioma Study Group (2006). Fluorescence-guided surgery with 5-aminolevulinic acid for resection of malignant glioma: a randomised controlled multicentre phase III trial. Lancet Oncol.

[20] Uckermann O, Yao W, Juratli TA, Galli R, Leipnitz E, Meinhardt M, Koch E, Schackert G, Steiner G, Kirsch M (2018). IDH1 mutation in human glioma induces chemical alterations that are amenable to optical Raman spectroscopy. J Neurooncol.

[21] Willems PWA, Taphoorn MJB, Burger H, Berkelbach van der Sprenkel JW, Tulleken CAF (2006). Effectiveness of neuronavigation in resecting solitary intracerebral contrast-enhancing tumors: a randomized controlled trial. J Neurosurg.

[22] Zhang L, Zhou Y, Wu B, Zhang S, Zhu K, Liu C-H, Yu X, Alfano RR (2021). Intraoperative detection of human meningioma using a handheld visible resonance Raman analyzer. Lasers Med Sci.

